# Food-Borne Transmission of Tick-Borne Encephalitis Virus—Spread, Consequences, and Prophylaxis

**DOI:** 10.3390/ijerph19031812

**Published:** 2022-02-05

**Authors:** Alicja M. Buczek, Weronika Buczek, Alicja Buczek, Joanna Wysokińska-Miszczuk

**Affiliations:** 1 Department of Biology and Parasitology, Faculty of Health Sciences, Medical University of Lublin, Radziwiłłowska 11 St., 20-080 Lublin, Poland; abuczek21@gmail.com (A.M.B.); wera1301@gmail.com (W.B.); 2 Chair and Department of Periodontology, Faculty of Medical Dentistry, Medical University of Lublin, Chodźki 6 St., 20-093 Lublin, Poland; periodontologia@tlen.pl

**Keywords:** tick-borne encephalitis virus, TBE outbreaks, food-borne transmission, milk-borne infections, alimentary infection

## Abstract

Tick-borne encephalitis (TBE) is the most common viral neurological disease in Eurasia. It is usually transmitted via tick bites but can also occur through ingestion of TBEV-infected milk and dairy products. The present paper summarises the knowledge of the food-borne TBEV transmission and presents methods for the prevention of its spread. The incidence of milk-borne TBE outbreaks is recorded in central, eastern, and north-eastern Europe, where *Ixodes ricinus*, *Ixodes persulcatus*, and/or *Dermacentor reticulatus* ticks, i.e., the main vectors of TBEV, occur abundantly. The growing occurrence range and population size of these ticks increases the risk of infection of dairy animals, i.e., goats, sheep, and cows, with viruses transmitted by these ticks. Consumers of unpasteurised milk and dairy products purchased from local farms located in TBE endemic areas are the most vulnerable to alimentary TBEV infections. Familial infections with these viruses are frequently recorded, mainly in children. Food-transmitted TBE can be monophasic or biphasic, and some of its neurological and psychiatric symptoms may persist in patients for a long time. Alimentary TBEV infections can be effectively prevented by consumption of pasteurised milk and the use of TBEV vaccines. It is recommended that milk and dairy products should be checked for the presence of TBE viruses prior to distribution. Protection of dairy animals against tick attacks and education of humans regarding the epidemiology and prophylaxis of TBE are equally important.

## 1. Introduction

Tick-borne encephalitis virus (TBEV) is one of the most important human tick-borne viral pathogens in Eurasia. It causes an acute febrile infectious disease, i.e., tick-borne encephalitis (TBE). In the last two decades, the spread of this virus has intensified considerably [1,2,3,4,5], and new foci have appeared in its current occurrence range [6,7,8,9].

Changes in the distribution of TBEV have contributed to a high increase in the incidence of human TBE in northern, central, north-eastern, and eastern Europe (e.g., [10,11,12,13,14,15,16,17,18,19]). Approximately 10,000–13,000 cases of this disease are registered each year, and the highest TBE incidence (>10/100,000 population) is reported in the Baltic countries, the Czech Republic, Russia, and Slovenia [8,17,20].

The majority of human TBEV infections are associated with ticks feeding on the host skin, but milk-borne transmission have also long been reported (e.g., [21,22,23,24,25,26,27,28,29,30,31,32,33]). Additionally, there have been sporadic cases of TBEV infection of patients undergoing transplantation of solid organs (kidney or liver) [34] and laboratory workers infected through the respiratory tract or oropharyngeal epithelial cells [35].

According to estimates, approximately 1% of all human TBEV infections are acquired through consumption of infected unpasteurised milk or dairy products from infected livestock [36,37]. It seems, however, that infections with this virus via this route may be more frequent. As in the case of virus transmission with tick saliva, some food-borne TBEV infections are asymptomatic or mildly symptomatic; hence, they may be undiagnosed and not included in statistics [38]. In as many as 30–50% of all recorded cases of human TBE in Germany, the patients did not remember the tick bite incident before the development of the disease, which suggests that some of the cases may have been caused by food-borne TBEV transmission [39].

Food-borne TBE can be diagnosed in humans, even in regions where the disease has not been previously reported. This may be the case of travellers who become infected with the virus present in milk or dairy products in a TBE endemic area before returning to their country [40].

The highest number of food-borne TBE infections is noted in Slovakia, where as many as 17% of all recorded human TBE cases are associated with the consumption of contaminated unpasteurised milk [41]. TBE outbreaks associated with the consumption of dairy products occur mainly in endemic areas of this disease. Unpasteurised milk is considered by some groups of consumers to have a higher nutritional value than pasteurised milk. As shown during a food-borne TBE epidemic in the Czech Republic, the major argument for drinking unpasteurised milk raised by consumers was the wish to provide a healthy diet for their family members [36]. In addition to the advocates of consumption of unpasteurised milk, producers as well as farm workers and their families are consumers of this type of milk [42]. A large group of consumers of unpasteurised dairy products are those who prefer cheese and other dairy products made from unpasteurised milk. Raw milk vending machines are popular in some regions where residents and tourists can buy local unprocessed dairy products [43].

The increase in global milk consumption may contribute to the spread of alimentary TBE virus infection [44,45]. For many people, especially children and the elderly, milk is the main source of many valuable nutrients, i.e., proteins, fats, carbohydrates, amino acids, minerals, vitamins (e.g., vitamin D), and somatic cells [46]. Milk also contains bioactive substances such as lactoferrin, i.e., an antimicrobial and antiviral protein [47,48], which can block mosquito-borne flavivirus receptor binding [49].

As many as 24 of the 30 countries with the highest consumption of milk and dairy products per capita are European countries [50]. They are represented by the Baltic countries (Finland, Sweden, Denmark, Norway, Lithuania, Estonia) as well as central (Switzerland, Austria, Germany) and south-eastern (Slovenia, Croatia, Greece) European countries, where an increase in the TBE incidence is reported.

Due to the possibility of human TBEV infection through the consumption of unpasteurised milk and dairy products, the present study focuses on the determinants of the development of tick-borne encephalitis of nutritional origin in humans, the epidemiology and symptoms of this disease, and methods for the prevention of food-borne transmission of this virus.

## 2. Circulation of Tick-Borne Encephalitis Viruses in Nature

TBEV is a small (diameter from 40 to 60 nm) single-stranded RNA virus [51,52] from the genus *Flavivirus*, representing the family *Flaviviridae*. Its virion consists of a nucleocapsid surrounded by a membrane composed of host-derived lipids in which the viral envelope and membrane proteins are embedded [53]. Protein E, i.e., the major antigen inducing the production of virus-neutralising antibodies, can be inactivated by pasteurisation [54].

Several TBEV subtypes occur in various geographic regions of Europe and Asia, mainly the European (TBEV-Eu), Siberian (TBEV-Sib), and Far Eastern (TBEV-FE) subtypes [55,56,57], and there are two TBEV variants, which are probably new subtypes, i.e., Baikalian (TBEV-Bkl) and Himalayan (TBEV-Him) [57,58,59,60,61].The European subtype causes a milder form of the disease, with a mortality rate of 0.5%-2% [17]. Infections caused by the TBEV-Sib and TBEV-FE subtypes are characterised by severe symptoms, and their mortality rates range between 5 and 20% [62].

Various abiotic (e.g., microclimatic conditions) and biotic factors (e.g., the presence of ticks—vectors, hosts of various tick developmental stages, and zoonotic reservoirs of the pathogen) have an impact on TBEV circulation in nature [10,16,63,64]. The main vectors of the virus are wide host-spectrum ticks such as *Ixodes ricinus* [65,66] and *Dermacentor reticulatus* [67,68] in various regions of eastern, central, western, northern, and southern Europe and *Ixodes persulcatus* in the north-eastern part of the continent [55,56,69,70]. TBEVs can probably also be transmitted by other species of ticks, e.g., *Dermacentor marginatus* [67,71] and *Haemaphysalis concinna* [72].

In the last few decades, climate and environmental changes have contributed to the considerable expansion of the occurrence range of ticks with great epidemiological importance [1,2,16,73,74,75] and an increase in their population size [76,77,78] in Eurasia. The duration of the activity of ticks with high vector competence, e.g., *Ixodes ricinus* and *Dermacentor reticulatus*, which can attack hosts in central Europe, even in the winter months when the temperatures exceed 0 °C and there is no residual snow layer [79,80,81]. Changes in tick phenology increase the risk of tick attacks on hosts, e.g., dairy animals grazing on pastures during the tick activity period.

The host may be infected with flaviviruses by ticks shortly after the attachment to the skin and onset of blood ingestion. Experimental studies have shown that the nymphs of *Ixodes scapularis* deer ticks can efficiently transmit Powassan flavivirus to naive mice 15 min after their hypostome is attached in the host skin [82].

## 3. Spread of Viruses in Dairy Animals, Milk, and Dairy Products

The infection of sheep, goats, and cattle with the TBE virus is usually asymptomatic, but the blood serum of these animals contains TBEV-specific antibodies [11,83,84,85,86]. Nevertheless, there are literature reports on symptomatic (and fatal) cases of livestock infection caused by this virus [87]. In goats and sheep, which are considered sentinel hosts of viruses, antibodies persisted even after 28 months, although there were no disease symptoms [88]. The seroprevalence of anti-TBEV antibodies in dairy animals varies across regions. For example, TBEV-specific antibodies were detected in 4.3% of the blood sera of 4114 goats examined in the Canton of Valais, Switzerland [89] and in 14.6% of 662 serum samples collected from goats from 37 flocks in a non-endemic region in the Canton of Ticino [88].

In north-western Romania, TBEV was identified with the use of the virus neutralisation test (VNT) in 15.02% of sheep in 80% of the 50 examined localities in five counties. In this area, the seroprevalence of TBEV in sheep ranged from 2% in Mureș County to 27.73% in Bihor County [90]. In total, 706 goat sera from 69 farms were screened for TBEV-specific antibodies, resulting in the detection of five positive farms in northeast Italy [4]. Substantial differences in seroprevalence, which ranged between 0% and 43%, were noted in 3590 sheep and 3793 goats examined in 2003 and 2006–2009 in various districts of Germany, i.e., Baden-Wuerttemberg, Bavaria, and Thuringia [91]. In Norway, specific antibodies to TBEV were detected in cows on only one (in Arendal) of five examined farms, but their prevalence was as high as 88.2% [92].

As confirmed by some studies, the seroprevalence in domestic animals correlates with the TBE incidence in humans [4,11,83,84].

During the viraemic phase of the infection, TBEV is secreted with the milk of dairy animals. The TBEV transmission into milk is probably supported by the immunosuppression of the animals caused by infection with another tick-borne pathogen, i.e., *Anaplasma phagocytophilum* [93]. An increase in the TBEV antibody response after TBEV and *A. phagocytophilum* co-infection in sheep was reported by Paulsen et al. [94].

As demonstrated in experimental studies, TBEV can be excreted in goat, sheep, and cow milk for approximately 3–7 days, starting from the second and third days after infection [95,96,97,98]. In other studies, TBEV was detected repeatedly in the milk of infected goats for 5–25 days after infection of these animals, and its virulence persisted in such dairy products as yogurt, cheese, and butter [56]. The TBEV virulence in the milk of infected cows, goats, and sheep persists for 8 to 19 days after infection [85,99].

TBEV RNA was identified in unpasteurised milk from 5.4% of cows tested on farms located in three municipalities of Mandal, Skedsmo, and Brønnøy (Norway) [92]. In the tick-borne encephalitis risk area in Lublin Province (eastern Poland), 119 unpasteurised milk samples were collected from 63 cows, 29 goats, and 27 sheep reared on eight farms. The highest TBEV prevalence was detected in the sheep (22.2%) and goat (20.7 %) milk, whereas the cow milk was characterised by a twofold lower prevalence (11.1%) [100]. In turn, in Baden-Württemberg (Germany), 22 cheese samples from goat milk from 18 different batches were tested, and five samples (21.7%) from five different batches of cheese (cream cheese, soft cheese, and ripened cheese) were RT-qPCR-positive for the TBEV-genome [39].

TBEV infections in farmed animals and, consequently, their milk are seasonal, which is related to the vector activity rhythms. In eastern and central Europe, numerous alimentary TBE outbreaks were recorded from May to July [33,101], i.e., during the spring peak activity of *I. ricinus*, *I. persulcatus*, and *D. reticulatus* ticks attacking animals grazing in pastures. In some areas, such as Slovakia, TBE outbreaks after consumption of cow, goat, and sheep milk/cheese were reported in October and November [101], i.e., during the autumn peak activity of adult *D. reticulatus* parasitizing on dairy animals in Central Europe.

TBEV antibody analysis of bulk tank milk samples collected on 554 dairy farms in Sweden revealed a higher incidence of TBEV antibodies in autumn (November) (4.8%) than in spring (May) (3.0%), which is associated with the increased activity of ticks in summer in this part of the continent, accompanied by an increase in the incidence of cow infection with viruses [102].

## 4. Alimentary Virus Transmission and Symptoms of Food-Borne TBEV Infections

TBEV infection in humans can occur through the consumption of unpasteurised milk from infected goats [33,36,39,98,100,101,103,104,105,106,107,108,109,110,111], sheep [26,100,101,112], and cows [29,92,100,102,113] (Table 1).

The mechanism of TBEV transmission in food has not been fully elucidated. The maintenance of the virulence of the virus may be influenced by the mode of digestion of dairy products in the gastrointestinal system. Milk remains in the stomach, where the pH is low, for a relatively short time, and its first portions leave the stomach and reach the duodenum within a few minutes. After 1.5 to 2 h, there is no longer milk in the stomach [70]. As confirmed by the research conducted by Pogodin [114], TBEVs maintain their infectivity in normal gastric juice (pH 1.49–1.80) for up to 2 h. This may explain why these viruses contained in ingested milk maintain their virulence until they penetrate the duodenum, where they probably penetrate the M cells of Peyer’s patches and then reach the lymphatic tissue. Thus, they may be released into the circulatory system and transferred to various organs, including the brain, with blood [85,115]. TBE outbreaks affect consumers of milk and dairy products originating from the same sources, e.g., those purchased directly from local producers [31,39,101,109,110,113] or in venues selling regional products, e.g., marketplaces and restaurants [33,101]. TBEV infections often affect members of the same family (e.g., [29,30,33,36,101,105,111,116]). In the Czech Republic, 33 TBE cases (51.6%) were detected as family outbreaks after consumption of cheese or milk purchased from animal breeders. In familial infections, children more often suffer from this disease, as they are probably the main consumers of contaminated milk [36].

One group at high risk of food-borne infection are shepherds grazing goats and/or sheep in the mountains and consuming unpasteurised milk and dairy products. Transmission of tick-borne encephalitis virus (TBEV) through unpasteurised goat milk in shepherds was described, e.g., in an alpine pasture located at 1500 m above sea level in western Austria [106].

Food-borne TBE, formerly referred to as biphasic milk fever, may be asymptomatic or may induce symptoms appearing once or twice with a remission period between their occurrences [106,110,117,118]. The monophasic TBE form was diagnosed in approximately 50% of 274 patients hospitalised after the consumption of unpasteurised contaminated milk from a local dairy during a milk-borne TBE outbreak in Rožňava (Slovakia) in 1951, during which over 600 subjects were infected by the virus [117].

The TBE incubation period in milk-borne infection may range from 2 days to 4 weeks (e.g., [108,111]). For example, during the epidemic in Croatia, Ilic et al. [111] found that TBE symptoms in most patients developed two weeks after consuming raw (unpasteurised) goat milk. In turn, Brockmann et al. [39] reported a shorter incubation period of 3 to 7 days in patients infected with the virus from unpasteurised goat milk and cheese in Baden-Württemberg. Dorko et al. [101] reported an average incubation period of 13.8 days (4 days–4 weeks) after consumption of goat, sheep, and cow milk/cheese.

The course of the disease and symptoms of alimentary infection with the virus may vary between patients [33,39,106,108,118]. In the first phase, nonspecific flu-like symptoms persist for ca. 3–7 days. They most often include an increase in body temperature to approx. 38.5 °C–40.0 °C, headache, limb weakness, nausea, and vomiting. After a ca. 8-day period of remission, there may be a second phase with high temperature, headaches, nausea, and vomiting as well as signs of meningeal irritation and/or encephalitis. In the mild course of the second phase, symptoms resolve after 3–4 days. In severe cases, they usually persist for 12–21 days, and sometimes even for several months [39,108]. Although the overall health of the patient may improve, some symptoms such as asthenia and neurological and psychiatric disorders may appear one year after food-borne TBEV infection [39].

Less common symptoms of alimentary TBEV infection were found to include disturbances in vision, blurred vision or diplopia, neurasthenia, vertigo, fatigue syndrome, lower limb paraparesis, cough, tetany, paraesthesia, cerebral syndrome, and insomnia [101,118]. Dorko et al. [101] reported the death of a 47-year-old man diagnosed with TBE, who may have contracted the virus via consumption of unpasteurised goat milk. However, because the patient also reported a tick bite incident in the history, it was not possible to determine the source of the TBEV infection clearly.

A comparison of the frequency of food-borne TBE cases in different patient groups has shown that the risk of infection in children aged 5 to 9 years is 2.5 times greater than that in adults aged from 35–44 to 75+ years [36]. No analyses have been performed to compare the disease symptoms in children and adults in alimentary TBEV infection. A relationship between the age of patients and the TBE symptoms and course was confirmed in cases of tick bite-transmitted TBEV infections. TBE was usually milder in children than in adults [37].

## 5. Methods for Prevention of Alimentary TBEV Infection and Determinants of Virus Survival in Food

Food-borne TBEV transmission can be effectively reduced by the consumption of pasteurised goat, sheep, and cow milk.

The International Dairy Foods Association [119] recommends that milk should be pasteurised by heating to 72 °C for 15 s and then immediately cooled to refrigeration temperatures (4 °C). Rónai et al. [120] found that both fast (72 °C, 15 s) and soft (63 °C, 30 min) pasteurisation destroyed viable TBE virus particles. As shown by these authors, in addition to pasteurisation, the addition of salt during cheese production can protect the consumer from TBEV infection.

While proper milk pasteurisation fully protects consumers against TBEV infection, temperature does not completely inactivate the virus in the milk used for cheese production [121]. Therefore, procedures for the reduction of the possibility of contamination of milk intended for direct consumption and all dairy products, including cheese, should be implemented in TBE endemic areas.

In unpasteurised milk from sheep and goats stored at a temperature of 4 °C, the TBE virus infectivity is preserved for at least 3 days [5]. Similar results were obtained by Offerdahl et al. [121] in experimental studies on the thermal stability of flaviviruses (BSL-2 TBFV) in unpasteurised goat milk. At room temperature and 37 °C, complete inhibition of TBE virus replication in colostrum and milk was found after 24 h and 48 h, respectively [5].

The risk of alimentary TBEV infections depends on the amount of viruses in milk and dairy products. In unpasteurised goat milk and unsalted cheese with a small amount of TBEV, virulent particles were detected for 5–10 days. In the case of larger amounts, virulent particles were present for up to 20–25 days in milk and for 10–15 days in unsalted cheese samples, regardless of the seasoning used [120].

Vaccination against TBEV is an effective prophylaxis of the development of TBE caused by tick- and food-borne infections [38,99,108,122,123,124,125]. The market provides different TBE vaccines, e.g., FSME-IMMUN^®^, Encepur^®^, EnceVir, and IPVE (also called TBE vaccine Moscow) vaccines containing inactivated strains of viruses, which protect against the European, Siberian, and Far-Eastern subtypes of TBEV [123,126,127]. According to a standard schedule, the vaccines are administered intramuscularly in three doses, usually with an interval of 1–3 and 5–12 months between the first and second and the second and third dose, respectively [128].

Because the knowledge of tick-borne diseases declared by the inhabitants of various regions is still unsatisfactory, education in the field of TBE epidemiology and prophylaxis in various groups of people, especially milk consumers, farmers, and dairy cattle breeders, may contribute to the reduction of the incidence of alimentary TBEV infections [129,130,131].

Reduction of the number of ticks, i.e., TBEV vectors, in TBE occurrence areas with the use of various chemical and biological methods (e.g., [132]) may reduce the exposure of dairy animals to tick infestations.

## 6. Conclusions

The expansion of the tick occurrence range and the increase in the tick population size have resulted in an increased risk of exposure of tick hosts, i.e., goats, sheep, and cows, to infection with TBE viruses, which are introduced with the saliva of ticks during blood ingestion. Consumption of unpasteurised milk and dairy products derived from infected dairy animals poses a risk of food-borne transmission of the virus to humans. The course of TBE disease, with possible long-term neurological symptoms, suggests the need to undertake various measures to reduce human exposure to its causal agent.

In order to reduce the incidence of alimentary TBEV infections, the consumption of pasteurised milk and its products should be recommended. Special attention should be paid to the methods for manufacturing dairy products (cheese, yoghurt) in local factories in TBE endemic areas, which should ensure full protection against TBEV contamination in goat, sheep, and cow milk.

Before distribution by local producers, unpasteurised dairy products should be analysed for the presence of viruses, and/or TBEV infected dairy animals providing milk for human consumption should be excluded. It is recommended that the prophylaxis of TBEV transmitted via food and by ticks should be based on the use of vaccines against TBEV available on the market. It is also indispensable to develop a strategy for controlling ticks (TBEV vectors) and for the protection of their potential hosts (dairy animals) against attacks by these arthropods.

## Figures and Tables

**Table 1:** Examples of tick-borne encephalitis (TBE) outbreaks in Europe related to milk and cheese consumption.

Country	Years/Months	Number of Ill/Exposed People/Outbreaks	The Source of the Infection (Number/% of Hospitalized Patients)	References
Slovak Republic	2012–2016/ April–November, the most outbreaks from April to June	110/714/13	goat’s milk, cheese (41 /37.3%) sheep’s milk, cheese (67/60.9%) cow’s milk, cheese (2/1.8%)	Dorko et al. 2018 [101]
Czech Republic	1997–2008	64/nd/nd	goats’milk (36/56.3%) sheep’s milk, cheese (21/32.8%) dairy milk (7/10.9%)	Kriz, Benes and Daniel 2009 [36]
Hungary	2007/August 2011/September–October	25/154/1 30/nd/nd	goat milk cow milk	Bologh et al. 2010 [107] Caini et al. 2012 [113]
Slovenia	2012/May	3/5/1	goat milk	Hudopisk et al. 2013 [108]
Croatia	2015/ April–May 2019/ June	7/10/1 5/nd/1	goat milk or cheese goat milk	Markonovović et al. 2016 [109] Ilic et al. 2020 [111]
Austria	2008	6/nd/1	self-made cheese from mixture of goat milk and cow milk	Holzmann et al. 2009 [106]
Germany	2016/May–June	2/3/1	goat milk, cheese	Brockmann et al. 2018 [39]
Poland	2017/June	4/nd/1	goat milk	Król et al. 2019 [110]
Estonia	2005/ May–June	27/nd/nd	goat milk	Kerbo et al. 2004 [33]

## Data Availability

Not applicable.
